# Differentiation Effects of Platelet-Rich Plasma Concentrations on Synovial Fluid Mesenchymal Stem Cells from Pigs Cultivated in Alginate Complex Hydrogel

**DOI:** 10.3390/ijms160818507

**Published:** 2015-08-07

**Authors:** Hao-Che Tang, Wei-Chuan Chen, Chih-Wei Chiang, Lei-Yen Chen, Yu-Ching Chang, Chih-Hwa Chen

**Affiliations:** 1 Department of Orthopedic Surgery, Chang Gung Memorial Hospital, Keelung 204, Taiwan; E-Mail: tanghaoche@gmail.com; 2 College of Medicine, Chang Gung University, Taoyuan 333, Taiwan; 3 Bone and Joint Research Center, Department of Orthopedics and Traumatology, Taipei Medical University Hospital, School of Medicine, College of Medicine, Taipei Medical University, Taipei 110, Taiwan; E-Mails: itispay@gmail.com (W.-C.C.); kiwi8502017@gmail.com (C.-W.C.); 4 Spine Department, Lin-Sun Hospital, Taichung 403, Taiwan; E-Mail: ryandddchen@hotmail.com; 5 Division of Pediatric General Medicine, Department of Pediatrics, Chang Gung Memorial Hospital, Taoyuan 333, Taiwan; E-Mail: Choelchang@gmail.com; 6 Graduate Institute of Biomedical Materials and Tissue Engineering, College of Biomedical Engineering, Taipei Medical University, Taipei 110, Taiwan

**Keywords:** platelet-rich plasma, synovial fluid mesenchymal stem cells, pigs, alginate hydrogel, gene expressions, DNA proliferation, chondrogenesis

## Abstract

This article studied the effects of platelet-rich plasma (PRP) on the potential of synovial fluid mesenchymal stem cells (SF-MSCs) to differentiate. The PRP and SF-MSCs were obtained from the blood and knees of pigs, respectively. The identification of SF-MSCs and their ability to differentiate were studied by histological and surface epitopes, respectively. The SF-MSCs can undergo trilineage mesenchymal differentiation under osteogenic, chondrogenic, and adipocyte induction. The effects of various PRP concentrations (0%, 20% and 50% PRP) on differentiation were evaluated using the SF-MSCs-alginate system, such as gene expression and DNA proliferation. A 50% PRP concentration yielded better differentiation than the 20% PRP concentration. PRP favored the chondrogenesis of SF-MSCs over their osteogenesis in a manner that depended on the ratios of type II collagen/type I collagen and aggrecan/osteopontin. Eventually, PRP promoted the proliferation of SF-MSCs and induced chondrogenic differentiation of SF-MSCs *in vitro*. Both PRP and SF-MSCs could be feasibly used in regenerative medicine and orthopedic surgeries.

## 1. Introduction

Both mesenchymal stem cells (MSCs) and platelet-rich plasma (PRP) have attracted a lot of attention in recent years because of their potential use in regenerative medicine and tissue engineering. MSCs are pluripotent cells that have the capacity for trilineage mesenchymal differentiation in response to appropriate signals [1]. MSCs have been isolated from a wide range of tissues, including bone marrow, periosteum, synovium, adipose tissue, trabecular bone, skeletal muscle and deciduous teeth [2]. In orthopedic medicine, the therapeutic application of MSCs in animal models involves spinal fusion, and the repair of articular cartilage, ligament, and meniscus [3,4,5,6].

Synovial fluid mesenchymal stem cells (SF-MSCs) are MSCs that are present in synovial fluid. Owing to their location, harvesting SF-MSCs is less invasive than harvesting other MSCs, such as bone marrow (BM), umbilical cord blood, and adipose tissue. Osteoarthritis [7,8], anterior cruciate ligament injuries [9], and meniscus injuries [10] increase the number of SF-MSCs in the knee, indicating that SF-MSCs might participate in tissue repair. These facts make SF-MSCs as a potential treatment for injuries around the joints.

PRP contains concentrated growth factors, including transforming growth factor beta (TGF-β), basic fibroblast growth factor (bFGF), platelet-derived growth factor (PDGF), epidermal growth factor (EGF), and vascular endothelial growth factor (VEGF) [11,12,13]. These growth factors may help to accelerate tissue healing [14]. In clinical practice, PRP has been utilized in treating chronic epicondylitis [15,16], chronic plantar fasciitis [17], anterior cruciate ligament reconstruction [18,19], and osteoarthritis [20,21].

Both SF-MSCs and PRP have the advantage of being autologous and easy to harvest, and they have been observed to have positive effects in clinical practice. However, the exact mechanisms how SF-MSCs and PRP work are not yet understood. Therefore, this study looks at the effects of PRP on SF-MSCs *in vitro*.

## 2. Results and Discussion

### 2.1. Results

#### 2.1.1. Phenotype of Autologous Synovial Fluid Mesenchymal Stem Cells (SF-MSCs) from Pigs

**Figure 1:**
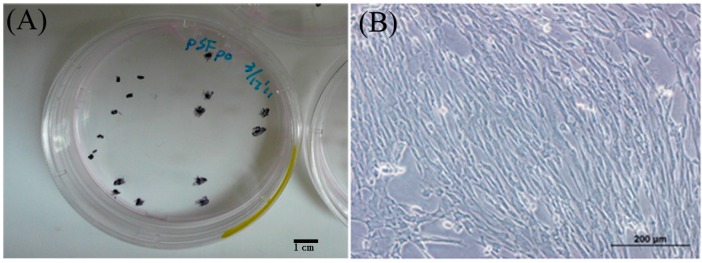
(**A**) Cell colonies of synovial fluid mesenchymal stem cells (SF-MSCs) after a 10-day cultivation; (**B**) The morphology of SF-MSCs. Three SF-MSCs samples were performed for this experiment.

Spindle-shaped SF-MSCs of pigs were observed using a light microscope; their phenotype is similar to that of human SF-MSCs [10]. Only a few approximate colonies are shown in passage 0 after 10 days of cultivation (Figure 1A). The SF-MSCs maintained their spindle shape for four passages (Figure 1B).

#### 2.1.2. Identification of Autologous SF-MSCs from Pigs

**Figure 2:**
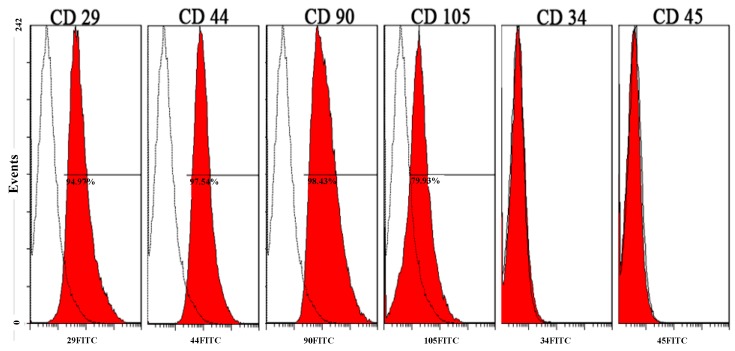
The identification of SF-MSCs by flow cytometry. When the area with red color was overlapped with the area without red color, it means the cluster of differentiation (CD) marker is not expressed. Three SF-MSCs samples were performed for this experiment.

Positive rates for cluster of differentiation 29 (CD 29), CD 44, and CD 90 in the SF-MSC population were close to or exceeded 90%. CD 105-positive rates in the same population exceeded 60%, whereas CD 34- and CD 45-positive rates were less than 5% (Figure 2). In conclusion, SF-MSCs expressed CD 29, CD 44, CD 90, and CD 105, but not CD 34 or CD 45. Accordingly, the characteristic immunophenotype of mesenchymal stem cells was verified [1].

#### 2.1.3. Potential of SF-MSCs to Differentiate

**Figure 3:**
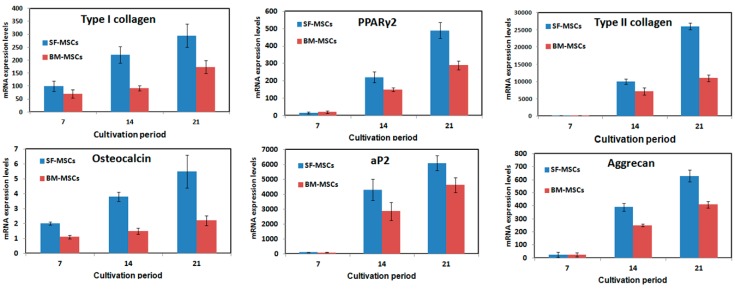

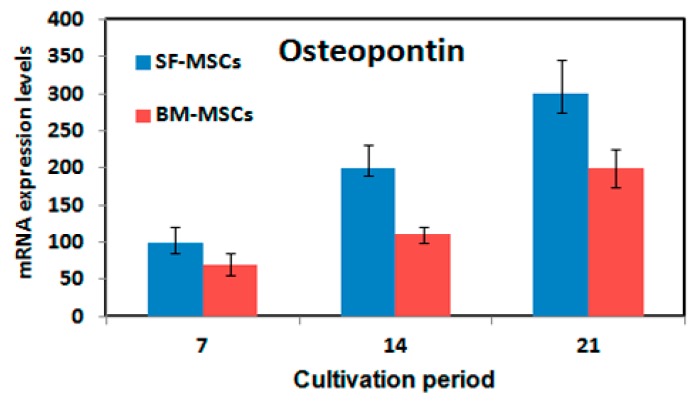
Evaluation of differentiation potential of SF-MSCs and bone marrow (BM)-MSCs by gene expressions. Gene expression profiles of SF-MSCs and BM-MSCs after osteogenic, chondrogenic, and adipogenic induction. Among them, type I collagen, osteocalcin, and osteopontin were for osteogenesis; type II collagen and aggrecan were for chondrogenesis; and peroxisome proliferator activated receptor γ 2 (PPAγ2) and adipocyte protein 2 (aP2) were for adipogenesis, respectively. Three SF-MSCs and BM-MSCs samples were performed for this experiment.

**Figure 4:**
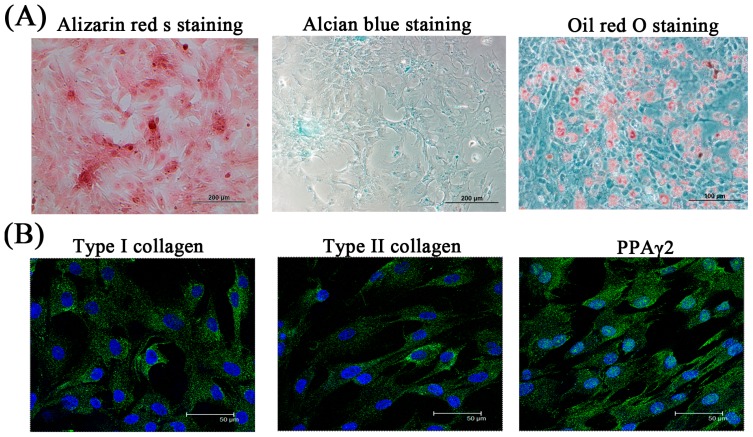
Evaluation of differentiation potential of SF-MSCs by histological staining and immunofluorescence staining. Histological staining (**A**) and immunofluorescence staining (**B**) of SF-MSCs after osteogenic (Alizarin red S stain and Type I collagen), chondrogenic (Alcain blue stain and Type II collagen), and adipogenic (Oil red O stain and PPAγ2) induction. Green: extracellular matrix and blue: cell nuclei.

The potential of SF-MSCs to differentiate was evaluated by histological staining and determination of marker gene expression (osteocalcin and osteopontin for osteogenesis, type II collagen and aggrecan for chondrogenesis, and peroxisome proliferator activated receptor γ 2 (PPAγ2) and adipocyte protein 2 (aP2) for adipogenesis), which were conducted in induction media. The results thus obtained reveal that all marker genes were upregulated in induction media during a one-to-three week cultivation period relative to the control group (cultivated without induction media) (*p* < 0.05). The expression of all marker genes by SF-MSCs exceeded that by BM-MSCs (*p* < 0.05, Figure 3). Histological staining and immunofluorescence staining were conducted to evaluate the potential of SF-MSCs to differentiate. The chondrogenic differentiation of SF-MSC yielded positive histological results for Alcian blue staining after medium induction (Figure 4A). Osteogenic differentiation assays revealed that most of the cells contained mineralized calcium deposits, as verified by Alizarin red S staining following medium induction (Figure 4A). The adipogenic differentiation of SF-MSCs was confirmed by Oil red O staining (Figure 4A). The results of the staining revealed that fats were secreted by SF-MSCs following medium induction. Immunofluorescence staining was also conducted to evaluate differentiation potential. As presented in Figure 4B, the cells (blue) were surrounded by a very large number of specific proteins following medium induction. Immunofluorescence staining yielded results similar to those concerning mRNA expression and those of histological staining. These experimental results concerning mRNA expression and those obtained by histological staining and immunofluorescence staining suggest that mesenchymal stem cells from synovial fluid can differentiate into cells of various types in various induction media *in vitro*.

#### 2.1.4. Effects of Platelet-Rich Plasma (PRP) Concentrations on SF-MSCs

The *in vitro* effects of PRP on the potential of SF-MSCs to differentiate were evaluated for osteogenesis and chondrogenesis. The PRP-SF-MSCs-Alginate (PSA) hydrogel system was utilized to evaluate cultivation by three PRP concentrations (0%, 20%, and 50%).

**Figure 5:**
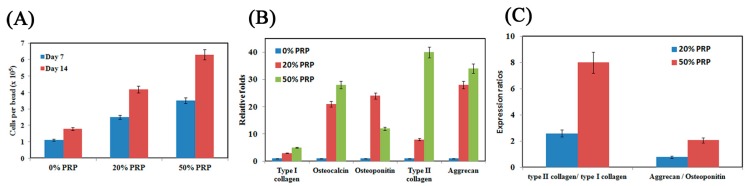
The differentiation potential effects of SF-MSCs among 0%, 20% and 50% platelet-rich plasma (PRP). Cell proliferation either on 7-day or on 14-day cultivation (**A**); gene expression after 14-day cultivation (**B**); and the ratios of type II collagen/type I collagen and aggrecan/osteopontin after 14-day cultivation (**C**). Three SF-MSCs samples were performed for this experiment.

As displayed in Figure 5A, cell proliferation was evaluated by the 3-(4,5-dimethylthiazol-2-yl)-2,5-diphenyltetrazolium bromide assay (MTT assay), which revealed that adding 50% PRP to the PSA hydrogel system promoted cell proliferation above that achieved in the either 20% or the control (0%) group (*p* < 0.05). This result reveals that PRP promoted SF-MSCs’ proliferation, which is consistent with a previous report [22].

**Figure 6:**
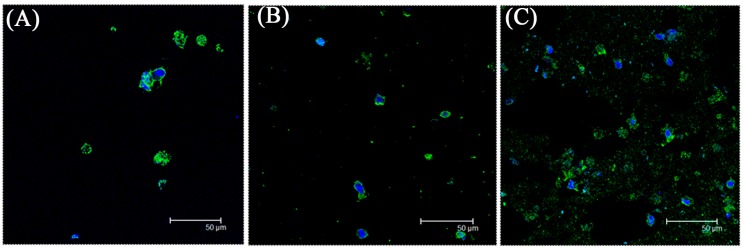
The immunofluorescence staining of type II collagen of SF-MSCs among 0% (**A**); 20% (**B**); and 50% PRP (**C**). The increased secretion of type II collagen was accompanied by the increase of PRP concentration. Green: type II collagen; blue: nuclei.

After 14 days of cultivation, the total RNA was harvested to evaluate gene expression. Figure 5B shows the gene expressions, which were normalized to the control group. Adding PRP to the alginate hydrogel upregulated all gene profiles for chondrogenesis-related genes (type II collagen and aggrecan) and osteogenesis-related genes (type I collagen, osteocalcin, osteopontin) (*p* < 0.05). The expression levels in all gene profiles, except for that of osteopontin, increased dose-dependently with PRP concentration during the cultivation period (*p* < 0.05). The 50% PRP concentration promoted differentiation more than did 20% PRP (*p* < 0.05). To confirm the effects of PRP on SF-MSCs’ differentiation, ratios of chondrogenesis-related genes to osteogenesis-related genes (type II collagen/type I collagen and aggrecan/osteopontin) were compared (Figure 5C). The differentiation was regarded as chondrogenesis if this ratio >1, and osteogenesis if this ratio <1. All of the ratios (type II collagen/type I collagen ratios were around 20 and aggrecan/osteopontin ratios were around 2) suggested that PRP stimulated SF-MSCs toward chondrogenesis rather than osteogenesis. Additionally, the immunofluorescence staining of type II collagen demonstrated that the 50% PRP concentration resulted in the secretion of more type II collagen than did 0% or 20% PRP concentration (Figure 6). This result is consistent with the profiles of chondrogenesis-related genes.

The concentrations of growth factor concentrations in PSA hydrogels (20% and 50%) were evaluated. The PRP in the PSA hydrogel system herein was activated using calcium ions to promote the release of growth factors [23,24]. The results thus obtained revealed that the concentrations of PDGF, bFGF, and TGF-β1 increased with the PRP volume (*p* < 0.05). Additionally, the concentration of TGF-β1 exceeded that of bFGF (Table 1, *p* < 0.05). According to the literature, bFGF, TGF-β1, and PDGF are associated with the osteogenesis, chondrogenesis, and proliferation of MSCs, respectively [25,26,27,28]. Therefore, the results herein may explain why PRP stimulated SF-MSCs toward chondrogenesis rather than osteogenesis.

**Table 1:** Concentrations of growth factors in PRP. Three PRP samples were performed for this experiment.

Growth Factors in PRP	TGF-β1 (ng/mL)	bFGF (pg/mL)	PDGF (ng/mL)
Before activation	2.34 ± 0.76	26.62 ± 3.13	11.56 ± 2.21
After activation (20%)	5.14 ± 0.36	30.91 ± 1.57	26.16 ± 1.91
After activation (50%)	10.12 ± 5.28	37.64 ± 5.33	34.53 ± 1.44
After activation (100%)	20.38 ± 7.08	51.32 ± 9.63	50.75 ± 2.76

### 2.2. Discussion

Independently of their origin, MSCs have both high proliferative potential and a multipotentiality for chondrogenic, osteogenic and adipogenic differentiation [1]. In 2001, De Bari *et al.* isolated synovial MSCs from human synovial membrane for the first time [29]. As well as having an ability to expand that is similar to that of bone marrow MSCs, human synovial MSCs exhibit greater chondrogenesis than those from other mesenchymal tissues [30]. SF-MSCs have been considered to derive from the synovium rather than bone marrow because they have similar characteristics to those of synovial MSCs, including morphology, colony size, and gene expression [9,10]. Jones *et al.* support this idea as they found a correlation between the presence of synovium clumps and the number of SF-MSCs [8]. The present study demonstrates that SF-MSCs can be easily harvested by arthrocentesis. The expression of the immunophenotype and the multidifferentiation potential in induction media verified that the obtained cells were mesenchymal stem cell-like cells.

Although the exact role of SF-MSCs in differential potential is not yet clear, SF-MSCs are thought to participate in intra-articular healing. According to Morito *et al.*, SF-MSCs in patients with an injured anterior cruciate ligament were 100 times more abundant than in healthy volunteers [10], and anterior cruciate ligament reconstruction surgery also increases the number of SF-MSCs. The authors observed that in a rabbit model, DiI-labeled synovial MSCs that were injected into the knee joint adhered to the rough surface of the anterior cruciate ligament. Based on the above findings, SF-MSCs may be associated with anterior cruciate ligament repair. In other studies, SF-MSCs have been claimed to be involved in cartilage repair. Jones *et al.* identified SF-MSCs in normal human and bovine synovial fluid from the knee joint, and found that the number of SF-MSCs was significantly increased in a knee with osteoarthritis [8]. Sekiya *et al.* further showed that the number of SF-MSCs increased with the radiological osteoarthritis grade [9]. In an animal model, synovial MSCs have been observed to enhance meniscus and cartilage regeneration [31,32].

PRP is an emerging biological tool in orthopedics and has been utilized clinically in the last decade. Percutaneous injection of PRP has successfully relived pain associated with chronic epicondylitis [15,16] and chronic plantar fasciitis [17]. In recent prospective studies, the intra-articular application of PRP for treating knee osteoarthritis and hip osteoarthritis has successfully relieved pain [20,21]. In a rabbit model of knee osteoarthritis, the intra-articular injection of PRP induced chondrogenesis and the generation of glycosaminoglycan [33,34]. Although the exact mechanism by which PRP operates remains unknown, concentrated growth factors in PRP are posited to participate in healing and to help recruit stem cells [11,12,13].

Owing to the favorable results of various clinical applications of PRP and the positive effects of SF-MSCs in many studies, the effect of PRP on SF-MSCs was evaluated herein. With an eye to future *in vivo* applications, the hydrogel system was used to eliminate the possibility of their being easily washed out with liquid and of the quick release of growth factors following activation by calcium. Our earlier study proved that SF-MSCs with PRP and thermosensitive hydrogel were very effective in cartilage regeneration and maturation. As published results have shown, 10% PRP in thermosensitive hydrogel can promote the repair of defective cartilage [35]. However, in our earlier study, extra animal experiments could not be conducted to verify the effects of the PRP concentrations owing to limitations on the number of available animals and funds. Therefore, in this study, *in vitro* experiments were conducted to explain the role of PRP in cartilage defect repair and to determine whether the effects are related to the concentration of PRP. As shown in Figure 4, PRP increased the proliferation of SF-MSCs, and the mitogenic effect was positively correlated with the concentration of PRP. Moreover, PRP upregulated both chondrogenesis-related genes and osteogenesis-related genes in a dose-dependent manner. Since chondrogenesis-related genes were upregulated more than osteogenesis-related genes, PRP was considered to stimulate chondrogenesis of SF-MSCs. These findings are consistent with the conclusion drawn by Mishra *et al.* [22], who found that PRP had a positive effect on the proliferation of human MSCs and directed MSC differentiation along a chondrogenic lineage. The reason for thus chondrogenic differentiation may be associated with the concentration of growth factors in PRP. In this study, the concentration of TGF-β1 in PRP was greater than that of bFGF. In the literature, TGF-β1 has been shown to promote the chondrogenesis of MSCs [26,27], bFGF has been shown to be associated with osteogenesis [25], and PDGF has been shown to have a critical role in the proliferation of MSCs [28]. Based on the above results, increasing the amount of PRP in hydrogel may increase its chondrogenic effect.

## 3. Experimental Section

### 3.1. Isolation of Autologous SF-MSCs from Pigs

All procedures performed on small-eared pigs were conducted according to the Guide for the Care and Use of Laboratory Animals and approved by the Committee of Experimental Animal Sciences of Chang Gung Memorial Hospital (identification code: 2009120910, 29 December 2009). Female, six small-eared pigs (weight 25 to 35 kg) were anesthetized with an intramuscular injection of a mixture of 1 mL of Rompun and 10 mL of ketamine. Ten milliliters of saline was injected into the knee joint with an 18G needle and 20-mL syringe, and then arthrocentesis was performed with the 18G needle and 20-mL syringe 30 s later. Under sterilized conditions, the combination of synovial fluid and saline was centrifuged at 1500 rpm for 5 min to separate the supernatant, and the pelleted cells were resuspended in Dulbecco Modified Eagle’s medium (DMEM, Gibco, Invitrogen Corp., Grand Island, NY, USA) supplemented with 10% fetal bovine serum (FBS, HyClone, Thermo Fisher Scientific, Waltham, MA, USA) and 1% antibiotics/antimycotics (10,000 U/mL penicillin, 10 mg/mL streptomycin, and 25 g/mL amphotericin B, PSA, Biological Industries, Kibbutz Beit-Haemek, Israel). The culture medium was replenished twice a week. The cell morphology was observed with a light microscope. The passage 4 of SF-MSCs was used in each experiment in this study.

### 3.2. Isolation of Autologous BM-MSCs from Pigs

All procedures performed on small-eared pigs were conducted according to the Guide for the Care and Use of Laboratory Animals and approved by the Committee of Experimental Animal Sciences of Chang Gung Memorial Hospital. Female, six small-eared pigs (weight 25 to 35 kg) were anesthetized with an intramuscular injection of a mixture of 1 mL of Rompun and 10 mL of ketamine. Ten milliliters of marrow were aspirated from the tibia using an 18-gauge needle, mixed with 1 mL of heparin (3000 units per mL) (Sigma, St. Louis, MO, USA) in a 15-mL centrifugation tube, and then washed with phosphate buffered saline (PBS) twice and the pelleted cells resuspended in DMEM supplemented with 10% FBS and 1% antibiotics/antimycotics (10,000 U/mL penicillin, 10 mg/mL streptomycin, and 25 g/mL amphotericin B, PSA). The culture medium was replenished twice a week. The passage 4 of MSCs was used in this study.

### 3.3. Identification of Autologous SF-MSCs from Pigs

For epitope profiling of the SF-MSCs, one million cells were suspended in 500 μL of PBS containing 20 μg/mL of antibody. After incubation for 30 min at 4 °C, cells were washed with PBS. Fluorescein isothiocyanate- or phycoerythrin-coupled antibodies against CD29, CD34, CD90, CD45, CD44, and CD105 (R&D systems, Minneapolis, MN, USA) were used. The SF-MSCs were supposed to express as the followings, CD 29^+^, CD 34^−^, CD 90^+^, CD 45^−^, CD 44^+^, and CD 105^+^ [1].

### 3.4. Autologous PRP Preparation

The PRP was collected by drawing whole blood from small-eared pigs via neck vessel penetration. Using a syringe with an 18-gauge needle, 9.8 mL of blood were aspirated and mixed with 0.2 mL of sterilized sodium citrate (55 mM, Sigma-Aldrich, St. Louis, MO, USA) for anticoagulation. The whole blood was then centrifuged at 1200 rpm (Eppendorf, Hamburg, Germany) for 10 min, and the superstratum formed the PRP. The numbers of platelets in the PRP and in the whole blood were counted respectively by a hemocytometer under a light microscope. The concentration of platelets in whole blood was (4.5 ± 0.8) × 10^8^ platelets/mL, and that in PRP was (1.9 ± 0.6) × 10^9^ platelets/mL (422% more in PRP than in whole blood).

### 3.5. Multipotent Differentiation

The *in vitro* multidifferentiation potential of the SF-MSCs and BM-MSCs toward osteogenesis, chondrogenesis, and adipogenesis was evaluated [36]. Osteogenic differentiation of MSCs was induced under the influence of β-glycerol phosphate, dexamethasone, and ascorbic acid. Chondrogenic differentiation was induced in MSCs mass cultures supplemented with 500 ng/mL recombinant human bone morphogenetic protein 2, 10 ng/mL transforming growth factor β3, 10^−7^ M dexamethasone, 50 g/mL ascorbate-2-phosphate, 40 g/mL proline, 100 g/mL pyruvate, and 50 mg/mL Insulin-Transferrin-sodium selenite (Sigma). Adipogenic differentiation was induced in expanded MSCs cultures by treatment with 1-methyl-3-isobutylxanthine, dexamethasone, insulin, and indomethacin.

### 3.6. Preparation of the PRP-SF-MSCs-Alginate (PSA) Hydrogel System

**Figure 7:**
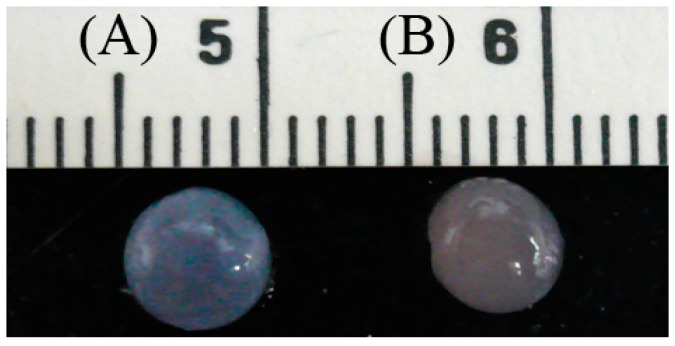
The morphology of PRP-SF-MSCs-alginate complex with (**A**) 0% PRP and (**B**) 20% or 50%. Scale unit: 1 cm.

Low-viscosity sodium salt alginic acid (Sigma) was used in these studies. This alginate is derived from *Macrocystitis pyrifera*, has a mannuronic acid/guluronic acid ratio of 1.67 and a molecular weight of around 50,000 Dalton. One milliliter of alginate (10%) was dissolved in 9 mL of PBS and sterilized for 30 min at 121 °C in an autoclave, then cooled at room temperature. PRP was added to the 1% alginate-PBS solution to form PRP-alginate solutions with 20% and 50% concentration of PRP, respectively, in a laminar flow hood. The SF-MSCs were treated with 0.5% trypsin before four passages, and a 5 × 10^4^ cells/mL density was prepared in the PRP-alginate solution. In addition, SF-MSCs were added into the alginate-PBS solution without PRP to make the control group. Then the PRP-SF-MSCs-alginate solution was dropped into a 0.1 M calcium chloride solution. After 2 min of reaction, the PSA hydrogel was obtained (Figure 7A,B). Then, the PSA hydrogels with different PRP concentration (0%, 20%, and 50%) were placed into 24-well culture plates, and 1 mL of DMEM medium (containing 10% FBS) was added to each well. The culture medium was replaced every two days.

### 3.7. Analysis of Gene Expression by Real-Time PCR

The cultured cells in the monolayer dish or PRP alginate hydrogels with the chondrogenic and osteogenic medium were analyzed by real-time PCR to investigate temporal mRNA expression changes. Total RNA was extracted from the cultured cells inside the monolayer dish for 1-week, 2-week, and 3-week cultivation, or PRP alginate hydrogels for 2-week cultivation. The monolayer dish or PRP alginate hydrogels were incubated with TRIzol reagent (Life Technologies, Grand Island, NY, USA). Subsequent steps were followed according to the manufacturer’s instructions. The total RNA of each sample was measured using a spectrophotometer (Beckman, Fullerton, CA, USA) and then was reverse transcribed with oligo-(dT) primers using Moloney Murine Leukemia Virus (MMLV) reverse transcriptase from a First Strand cDNA Synthesis Kit (Fermentas, Glen Burnie, MD, USA). The resultant solution (2.0 μL) was amplified in triplicate by real-time PCR (Applied Biosystems, Foster, CA, USA) with SyBr Green Master Mix reagent (Applied Biosystems). Glyceraldehyde phosphate dehydrogenase (GAPDH) was also amplified under the same conditions as a control. The primers for the specific genes were designed according to the published sequences available in GenBank using Primer Express 2.0 (Applied Biosystems) as shown in Table 2. The comparative Ct method was used for gene expression quantification.

**Table 2:** Sequences of primers used in real-time PCR.

Gene Symbol	Primer Sequences (3′→5′)	Size (bps)
GAPDH	AAGGGCATCCTGGGCTACAC GGTCCAGGGGCTCTTACTCC	230
Type I Collagen	AGAGGAGGGCCAAGAAGAAG ACGTCATCGCACAACACATT	174
Type II Collagen	TCTTCCTGGACCCGCCGGAC GCCACGCTCCCCTGGGAAAC	199
Osteocalcin	AACCCCGACTGCGACGAGCT ACACTTGCCGGGCAGGGAAG	174
Osteopontin	ACTCCGATGAATCCGATGAG TCCGTCTCCTCACTTTCCAC	220
Aggrecan	TGGCCACACATCGGGGTTGG GGCACTCGTCAAAGTCTGCCGT	239
aP2	AGGTTACGGCTTCTTTCTCACCTTG TTGGCCATGCCAGCCACCTT	214
PPAγ2	GCGCCCTGGCAAAGCACT TCCACGGAGCGAAACTGACAC	221

### 3.8. 3-(4,5-Dimethylthiazol-2-yl)-2,5-diphenyltetrazolium bromide assay (MTT) Assay

Cell proliferation was measured by MTT assays on days 0, 7, and 14 (Molecular Probes, Eugene, OR, USA). Briefly, SF-MSCs in PRP alginate hydrogels were cultivated in 96 wells first, then 2 mL of medium containing 0.5 mg/mL MTT was added into each culture well and incubated for 4 h in a 5% CO_2_ incubator at 37 °C. Consequently, the formed formazan crystal was dissolved by adding 2 mL of MTT solubilization solution. The solution was then transferred into 1.5-mL cuvettes, and the absorbance at 570 nm was taken three times.

### 3.9. Evaluation of Concentrations of Growth Factors in PRP

After PRP was harvested, the TGF-β1 (ng/mL), bFGF (ng/mL), and PDGF were evaluated by Porcine TGF-β1 Elisa kit (CUSABIO, Wuhan, China), Porcine bFGF Elisa kit (CUSABIO), and Porcine PDGF Elisa kit (CUSABIO) according to the manufacturer’s protocols. Briefly, 100 μL of sample and standard were added to each well for 1-h incubation at room temperature. After washing three times, 100 μL of the working dilution of the detection antibody was added to each well for 1-h incubation. After washing three times again, 100 μL of the working dilution of detection agent was added to each well for incubation for 20 min. Finally, 100 μL of the substrate solution was added to each well for 5-min incubation and then stop solution was added. The optical density of each well was taken immediately, using an enzyme-linked immunosorbent assay (ELISA) reader set to 450 nm. The values of TGF-β1, bFGF, and PDGF were shown in Table 1. One hundred percent PRP is used as an evaluation standard for growth factor quantification to compare 20% and 50% of PSA hydrogels.

### 3.10. Histological Staining and Immunofluorescence Staining

The detailed protocols of histological staining, *i.e.*, Alizarin red S staining, Alcian blue staining, and Oil red O staining, are according to the standard protocols. Briefly, SF-MSCs cultivated in chamber slides were fixed in 10% neutral buffered formalin. Alizarin red S staining was used to identify calcium deposition for osteogenesis. Alcian blue staining (Sigma) was used for proteoglycans detection for chondrogenesis. Oil red O staining was used to identify lipids and fats for adipogenesis. For immunofluorescence staining, SFMSCs in the PSA hydrogel was fixed in 10% neutral buffered formalin and embedded in paraffin. Then the sections of 5 μm were cut and collected on slides. Following permeabilization with 0.2% Triton X-100 (USB Corp., Cleveland, OH, USA) and blocking solution treatment (5% non-fat milk in PBS with 0.1% Triton X-100) for 30 min at room temperature, the slides were incubated with mouse anti-porcine monoclonal primary antibodies of type I collagen, type II collagen, and PPAγ2 (Merck Millipore, Darmstadt, Germany), applied for 1.5 h at room temperature. Subsequent to being washed three times with 1% non-fat milk in PBS with 0.1% Triton X-100, the slides were treated with Alexa 488 anti-mouse immunoglobulin G1 secondary antibody (Invitrogen Life Technologies, Carlsbad, CA, USA) for 45 min at room temperature. Finally, the samples were mounted using a mounting solution with DAPI (Life technologies, Grand Island, NY, USA) and left to dry overnight prior to observation.

### 3.11. Statistical Analysis

Each experiment was replicated three times. The data were expressed as means ± standard deviation (SD) by three experiments (*n* = 3) for each test. The control and experimental groups were compared to each other by a *t*-test.

## 4. Conclusions

In conclusion, both PRP and SF-MSCs have the advantage of being autologous and easier to harvest. Compared with recombinant growth factors or other products from animals, PRP is safer in terms of immunological reaction and less expensive. In this study, we found that PRP could enhance proliferation of SF-MSCs and induce chondrogenic differentiation of SF-MSCs *in vitro*. All of these make PRP and SF-MSCs potential tools in regenerative medicine and orthopedic surgeries.
